# Simple In-liquid Staining of Microbial Cells for Flow Cytometry Quantification of the Microbial Population in Marine Subseafloor Sediments

**DOI:** 10.1264/jsme2.ME21031

**Published:** 2021-08-24

**Authors:** Fumiaki Mori, Tomoya Nishimura, Taisuke Wakamatsu, Takeshi Terada, Yuki Morono

**Affiliations:** 1 Geomicrobiology Group, Kochi Institute for Core Sample Research, Japan Agency for Earth-Marine Science and Technology (JAMSTEC), Monobe B200, Nankoku, Kochi 783–8502, Japan; 2 Agricultural Science, Graduate School of Integrated Arts and Sciences, Kochi University, Monobe B200, Nankoku, Kochi 783–8502, Japan; 3 Marine Works Japan Ltd., Oppama-higashi 3–54–1, Yokosuka 237–0063, Japan

**Keywords:** cell counts, flow cytometry, sediment, SYBR Green I, microbial cell, marine sediment

## Abstract

Microbial cell counting provides essential information for the study of cell abundance profiles and biogeochemical interactions with the surrounding environments. However, it often requires labor-intensive and time-consuming processes, particularly for subseafloor sediment samples, in which non-cell particles are abundant. We developed a rapid and straightforward method for staining microbial intracellular DNA by SYBR Green I (SYBR-I) to enumerate cells by flow cytometry (FCM). We initially examined the efficiency of microbial cell staining at various dye/sediment ratios (volume ratio of SYBR-I/sediment [^v^SYBR/^v^Sed]). Non-cell particles in sediment strongly and preferentially adsorbed SYBR-I dye, resulting in the unsuccessful staining of microbial cells when an insufficient ratio (<1.63 ^v^SYBR/^v^Sed) of SYBR-I dye was present per volume of sediment. SYBR-I dye at an abundance of 10 ^v^SYBR/^v^Sed successfully and stably stained microbial cells in green fluorescence, while the fluorescent color of non-cell particles red-shifted to yellow-orange with the overaccumulation of SYBR-I dye. A low ^v^SYBR/^v^Sed ratio was quickly recognized by a colorless supernatant after centrifugation. At the appropriate ^v^SYBR/^v^Sed ratio, FCM-measured cell concentrations in subseafloor sediments were consistently similar to microscopy counts (>10^6^ cells cm^–3^). Samples with low cell abundance (<10^5^ cells cm^–3^) still require cell separation. This modified staining allows us to efficiently process and perform the microbial cell counting of sediment samples to a depth of a few hundred meters below the seafloor with a higher throughput and capability to scale up than procedures employing microscopy-based observations.

Marine sediment covers ~70% of the Earth’s surface. It harbors a remarkable microbial population that comprises 12–45% of the total microbial biomass or ~0.6–2% of the total living biomass on Earth (Bar-On *et al.*, 2018; Magnabosco *et al.*, 2018). Although the extent of the subseafloor biosphere has not yet been discovered, successive record-breaking discoveries in deep (to approximately 2.5‍ ‍km below the seafloor, Inagaki *et al.*, 2015) and high temperature (up to 120°C, Heuer *et al.*, 2020) zones of the subseafloor have expanded our understanding of habitable space in the subseafloor biosphere environment. Even though the subseafloor biosphere is characterized as a highly challenging habitat to survive, it holds diverse microbial communities matching soil and seawater and viable forms of microbial cells (Morono *et al.*, 2011b; 2020; Heuer *et al.*, 2020; Hoshino *et al.*, 2020). To gain further information on the geographical distribution and physicochemical control of the microbial community, it is of fundamental interest to accurately profile the abundance of microbes in marine sediment in a high-throughput manner.

Microbial cell counting in marine sediment has conventionally been conducted by eye-based enumeration with epifluorescence microscopy (EFM) using the fluorescent staining of microbial cells (Parkes *et al.*, 2000; Kallmeyer *et al.*, 2012). The fluorescence color-based approach to distinguish cells from non-cell particles overcame the methodological challenge of objective discrimination (Morono *et al.*, 2009). To enhance detection sensitivity, techniques that detach and separate cells from sediment particles worked well to reduce non-cell particles and concentrate cells from a higher volume of sediment for observations (Morono *et al.*, 2009; 2013; Kallmeyer *et al.*, 2012). Furthermore, by employing extensive measures to avoid procedural contamination during the sample handling process, cells down to <10 cells cm^–3^ in sediment (Morono and Inagaki, 2016) are detectable and countable. However, the entire procedure is often labor-intensive and time-consuming, particularly for subseafloor sediment containing finer particles and fewer cells than that in the shallow biosphere (Kallmeyer *et al.*, 2012).

An alternative approach is the use of flow cytometry (FCM), which is a powerful tool for identifying and enumerating fluorescent-labeled cells based on size, fluorescence intensity, and wavelength. FCM is commonly used in medical sciences and has been employed to study the ecology of microbial communities in various aquatic environments (Wang *et al.*, 2010; Irvine-Fynn *et al.*, 2012). However, it was only recently applied to sediment samples because of heavy analytical interference by abundant non-cell particles. Morono *et al.* (2013) previously demonstrated the applicability of their staining procedure to sample staining for FCM. They stained sediment slurry and density-separated cell concentrates, successfully distinguished microbial cells in FCM signals, and obtained EFM-comparable counts in a range of 10^4^ to 10^8^ cells cm^–1^.

However, as reported in previous studies (Morono *et al.*, 2013; Frossard *et al.*, 2016; Deng *et al.*, 2019), cell staining for FCM is not straightforward. The ratio of the cell count examined by FCM and EFM often varies with the amount of the sample applied (Morono *et al.*, 2013) or the condition of the sample, such as the organic matter content (Frossard *et al.*, 2016) and grain size (Deng *et al.*, 2019). The inaccuracy of the FCM method is generally attributed to insufficient sample staining and the resulting failure to recognize microbial cells separately from non-cell particles. The staining of cells on membranes provides brighter and more stable images (Morono *et al.*, 2013; Deng *et al.*, 2019), but involved a labor-intensive and one-by-one (difficult to scale up to a larger number of samples) process. Although Deng *et al.* (2019) optimized the staining procedure in a liquid suspension (“in-liquid staining” in this study), it still required a sample-by-sample optimization process.

We herein describe a direct and straightforward in-liquid staining method and the FCM quantification of microbial cells in subseafloor sediment. The results obtained revealed that the ratio of the dye to the sample is a key factor, and when this ratio is correct, direct in-liquid staining provides stable counts that are consistent with EFM counts. We also showed that the color of the supernatant after staining instantly indicates the success of staining. We applied our new staining procedure and counted microbial cells with FCM. The counts obtained were all similar to EFM counts in 10^6^ to 10^8^‍ ‍cells‍ ‍mL^–1^. Based on the present results, we discussed the applicability of this method and general recommendations.

## Materials and Methods

### Sediment sample collection

Core samples of marine subsurface sediment were collected during Integrated Ocean Drilling Program (IODP) expedition 329 in the South Pacific Gyre (Sites U1366, U1368, and U1371) in 2010 (Expedition 329 Scientists, 2011) and expedition 346 in the Japan Sea (Site U1428) in 2013 (Tada *et al.*, 2015) by the drilling vessel JOIDES Resolution. The sediment cores collected were immediately subsampled on board at depth intervals of 5‍ ‍cm for microbial analyses. Approximately 2‍ ‍cm^3^ of sediment samples for microbial cell counting was immersed in 8‍ ‍mL of 10% formalin solution and stored at 4°C.

### In-liquid staining for FCM quantification of cells: comparison of FCM cytograms with various dye amounts

In the comparison experiment of the dye amount gradient, cell staining was performed with six different amounts of SYBR Green I (SYBR-I, 10,000×; Thermo Fisher Scientific). To represent the amount of SYBR-I used to stain cells in sediment suspensions, we used the ratio of the volume (μL) between SYBR-I (10,000×) and sediment; when we stained 20‍ ‍μL of slurry, including 0.2‍ ‍μL of sediment, with 2‍ ‍μL of SYBR-I, the ratio was 10 SYBR-I/sediment volume (^v^SYBR/^v^Sed).

Sediment slurry (25‍ ‍μL, including 10‍ ‍μL of sediment) was diluted with 375‍ ‍μL of Tris-EDTA (TE) buffer (10‍ ‍mM Tris-HCl and 1.0‍ ‍mM EDTA, pH 8.0), and 50‍ ‍μL of detergent mix (100‍ ‍mM EDTA, 100‍ ‍mM sodium pyrophosphate, and 1% [v/v] Tween 80) and 50‍ ‍μL methanol were then added. Sediment slurry was sonicated (Bioruptor UCD-250; Sonicbio) in an ice bath for 20 cycles of 30‍ ‍s on at 200 W and 30‍ ‍s off. Slurry (20‍ ‍μL, including 0.2‍ ‍μL of sediment) was transferred to a new tube and stained with six different SYBR-I ratios (10, 5, 1.63, 0.63, 0.1, and 0.01 ^v^SYBR/^v^Sed) in the dark for 20‍ ‍min. Stained slurry was centrifuged at 5,000×*g* for 5‍ ‍min. After the supernatant had been removed and the volume adjusted to 10‍ ‍μL, 790‍ ‍μL of TE buffer and 200‍ ‍μL of custom-made beads solution (FluoSpheres Carboxylate-Modified Microspheres, 1.0‍ ‍μm, Green [505/515‍ ‍nm] and Deep Red [633/660‍ ‍nm], 2.66×10^5^ beads mL^–1^; Invitrogen) were added as an internal standard for volumetric calibration. After re-sonication in an ice bath for five cycles of 30‍ ‍s on at 200 W and 30‍ ‍s off, the sample (sediment sample final concentration of 0.2‍ ‍μL mL^–1^) was sieved with a 35-μm mesh to remove large particles and prevent clogging of the flow cell in the cytometer. It was then directly analyzed using a Gallios flow cytometer (Beckman Coulter) at a flow speed of 10‍ ‍μL min^–1^ and acquisition time of 3 or 10‍ ‍min, with FL1 as the channel for the triggering threshold (value=1 at the linear scale), a maximum acquisition event of 1.5×10^6^, and an average event rate of approximately 5,000 events s^–1^. Fluorescence detected in the FL1 (green fluorescence, 525/540‍ ‍nm), FL3 (orange fluorescence, 620/30‍ ‍nm), and FL4 (red fluorescence, 695/30‍ ‍nm) channels was used in data analyses with Kaluza analysis software (Beckman Coulter). Logarithmic dot plots of FL1/FL4 and FL3/FL4 were used to distinguish the signals of stained cells and fluorescent beads, respectively, from the background noise of non-cell particles. A FCM analysis of all samples was performed on the day of sample preparation. In the preparation of blank samples, filter-sterilized water (pore size of 0.2‍ ‍μm) was used instead of sediment slurry, which was processed by the same protocol described above.

### Re-staining in-liquid for FCM quantification of cells

To examine the effects of re-staining on inadequately stained sediment samples, samples (U1428A-1H1) subjected to the staining (SYBR-I ratio: 5, 1.63, 0.63, 0.1, and 0.01 ^v^SYBR/^v^Sed) and centrifugation (5,000×*g* for 5‍ ‍min) processes in the liquid staining method were re-stained with an additional 1‍ ‍μL of SYBR-I for 20‍ ‍min in the dark. Samples were then centrifuged, had the supernatant removed, were mixed with beads solution, sonicated, sieved, and then subjected to the FCM analysis, as described above.

### Membrane staining for the EFM cell count

Some of the stained sediment slurry (200‍ ‍μL) prepared for FCM was used for membrane staining and EFM counting, as described by Morono *et al.* (2009) with slight modifications. Approximately 3‍ ‍mL of filtered (0.22‍ ‍μm) 2.5% NaCl solution and 200‍ ‍μL of stained sediment slurry were placed into the filter tower and filtered using a black polycarbonate membrane with a pore size of 0.22‍ ‍μm (EMD Millipore). After air-drying, half of the membrane was re-stained by placing the filter on a drop of 80‍ ‍μL of SYBR-I solution (1/40‍ ‍[v/v] SYBR-I in TE buffer). After washing with 1‍ ‍mL of 50‍ ‍mM Tris buffer, the stained filter was mounted on a glass microscope slide with 6‍ ‍μL of mounting solution (2:1 mixture of VECTASHIELD mounting medium H-1000 [Vector Laboratories] and TE buffer). Fluorescence image acquisition (at 525/536‍ ‍nm [center wavelength/bandwidth] and 605/652‍ ‍nm by 490‍ ‍nm excitation) and cell enumeration were performed using an automated fluorescent microscope system (MetaMorph software; Molecular Devices), as described by Morono *et al.*, (2009). A cell separation technique was performed for the cell counting of sediment samples with less than 10^5^ cells cm^–3^, and cells were counted by eye-based enumeration using EFM, as described by Morono *et al.* (2013). To prepare blank samples, the same process was applied to the blank slurry prepared for the FCM analysis, and the stained filter was inspected by visual observations using EFM.

### Statistical analysis and visualization

Statistical analyses and visualization steps were performed in R (version 4.0.4; R Core Team, 2020). In the visualization steps, the “ggplot2” (Wickham, 2016) and “patchwork” (Pedersen, 2020) packages were used.

## Results and Discussion

### Effects of the amount of SYBR-I on the stainability of microbial cells in sediment samples for the FCM analysis

A previous study demonstrated that the staining of microbial cells in sediment samples was challenging because of the preferential adsorption of SYBR-I dye to non-cell particles, which take up DNA-stainable SYBR-I dye (Morono *et al.*, 2009). The addition of a high concentration (250-fold higher than the manufacturer’s recommendation) of SYBR-I dye to EFM counting membranes improved cell staining efficiency (Morono *et al.*, 2009). In FCM counting, the staining of microbial cells in sediment is successfully achieved using an on-membrane staining procedure that is optimized to obtain a stained cell suspension for counting (membrane trapping and staining on a membrane, followed by resuspending by ultrasonication). Although staining efficiency is sufficiently high to stain microbial cells and produce EFM-comparable counting results, it requires multiple steps and labor-intensive processes. On the other hand, in-liquid staining, in which SYBR-I dye is directly added to the sample suspension, is more straightforward and easier to perform. However, the stability of cell staining in our trials and in a previous study was insufficient (Deng *et al.*, 2019).

**Fig. 1.**
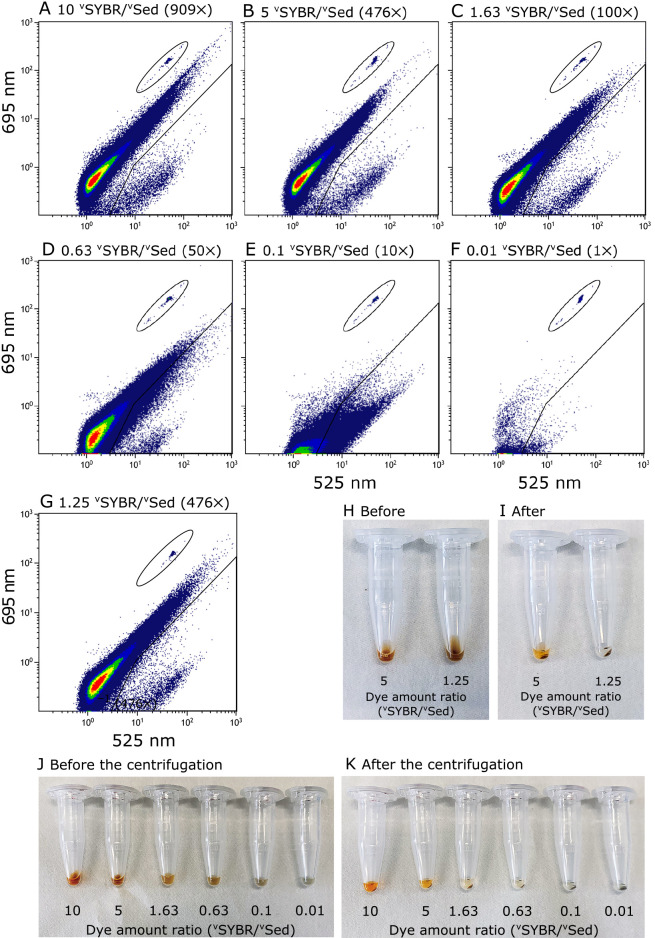
(A, B, C, D, E, F, and G) FCM cytograms of a sediment sample (U1428A-1H1) stained with various amounts of SYBR-I [(A) 10 ^v^SYBR/^v^Sed, (B) 5 ^v^SYBR/^v^Sed, (C) 1.63 ^v^SYBR/^v^Sed, (D) 0.63 ^v^SYBR/^v^Sed, (E) 0.1 ^v^SYBR/^v^Sed (F) 0.01 ^v^SYBR/^v^Sed, and (G) 1.25 ^v^SYBR/^v^Sed]. In (G), SYBR-I concentration (476×) was same as (B), but the sediment volume was fourfold higher than that of (B). The lower right portion below the solid line shows the region of the cell. The circle indicates the dots of beads. All cytograms show approximately 1,500 dots of beads, except (F), showing 1,045 dots of beads. (H, I, J, and K) Stained sediment suspension of FCM cytograms (A, B, C, D, E, F and G), before (H and J) and after centrifugation (I and K), respectively.

**Fig. 2.**
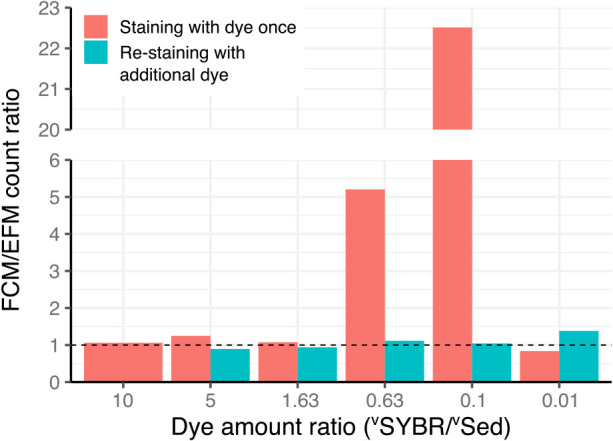
Bar plot of the ratio of the cell count between FCM and EFM. The dashed line indicates a FCM/EFM ratio=1.

We performed a gradient experiment on the amount of SYBR-I dye in the same volume of sediment samples (U1428A-1H1); different amounts of SYBR-1 were added to 20‍ ‍μL of the sediment suspension. Fig. 1 shows a scatter plot of the FCM-measured fluorescence intensity (cytogram) of samples stained using the in-liquid staining procedure with different amounts of SYBR-I dye. Two distinctive groups of dots representing cellular and non-cellular signals in the plot of green (525‍ ‍nm) and red (695‍ ‍nm) fluorescence were visible when staining was successful, as shown in Fig. 1A (green and yellow circles, respectively). However, when a lower amount of SYBR-I was applied for staining (Fig. 1D, E and F), all signals fell into the green region (lower right position in the graph), and no distinctive cellular region was visible in the plot. Furthermore, even the total number of detected signals (green fluorescence particles) decreased (reduction of 10.5% in Fig. 1F compared with that in Fig. 1A and Table S1), whereas the “real” total number of particles in the stained suspension was unchanged (*i.e.*, the only difference was the SYBR-I concentration). These results clearly demonstrated that SYBR-I dye did not stain non-cell particles. The overaccumulation of SYBR-I dye on non-cell particles shifted fluorescence color. However, when the accumulation of SYBR-I was inadequate, particles showed green fluorescence similar to microbial cells, which resulted in the significant overestimation of cell numbers (Fig. 2). A unique and complex issue in FCM-based analyses is all signals being shown as small dots in a 2D plot without any visual information. Therefore, FCM counts are more susceptible to unsuccessful staining than microscopic investigations, and it is important to establish whether cell staining is successful. We expressed the amount of SYBR-I dye applied to staining as the volume ratio of SYBR-I concentrate solution (10,000× concentrated, according to the manufacturer’s instructions) and sediment (^v^SYBR/^v^Sed [v/v]) needed for stable and successful staining. SYBR-I concentrations have been widely used to describe staining conditions. However, as shown in Fig. 1B and G, differences were observed in staining results based on the amount of sediment in suspensions even with the same concentration of SYBR-I. The region of sediment in Fig. 1G shifted towards a lower right position than in Fig. 1B. In comparisons with the results shown in Fig. 1C, in which staining was performed at a similar ^v^SYBR/^v^Sed ratio (1.63 in Fig. 1C and 1.25 in Fig. 1G), the cytograms obtained were similar. The concentration of adsorbates only controls adsorption. The volume (more precisely, the surface area) of adsorbents (*i.e.*, non-cell particles in the present study) ultimately controls the amount of SYBR-I dye available to stain microbial cells. Therefore, the ^v^SYBR/^v^Sed ratio is the most appropriate strategy for expressing the SYBR-I staining condition of sediment.

In terms of the accuracy of microbial cell counting, we compared FCM counts with EFM counts (Fig. 2). It is important to note that the FCM count at a ^v^SYBR/^v^Sed ratio of 1.63 was similar to the EFM count; however, as shown in Fig. 1C, some of the non-cell particle region was already in the cellular region, and the mixing of cellular and non-cellular signals occurred. This data error is easily overlooked in routine analyses and needs to be avoided by applying a higher ^v^SYBR/^v^Sed ratio. Therefore, a ^v^SYBR/^v^Sed ratio of at least 5, preferably 10, is recommended for staining in suspensions based on variations in the grain sizes of sediments of different lithologies.

We also discovered a simple indicator for estimating success/failure in the staining process: the color of the supernatant of staining slurry after a brief centrifugation. The color of the supernatant was clear in Fig. 1H, I, J, and K, which indicated that cells in the suspension were not properly stained, and non-cell particles also showed greener fluorescence. The further addition of SYBR-I dye (1‍ ‍μL of concentrated solution) worked well to stain cells in the suspension (Fig. 2).

In the present study, we performed FCM analyses on the same day as SYBR staining. FCM analyses of samples stored at 4°C for 2 days showed a ~20% increase in detected cells (data not shown), indicating a change in the fluorescent color of non-cell particles (yellow to green) due to the release of SYBR-I dye onto the surface of non-cell particles. Based on these results, FCM analyses need to be conducted on the day of sample staining.

### Application to sediment with various cell concentrations

**Fig. 3.**
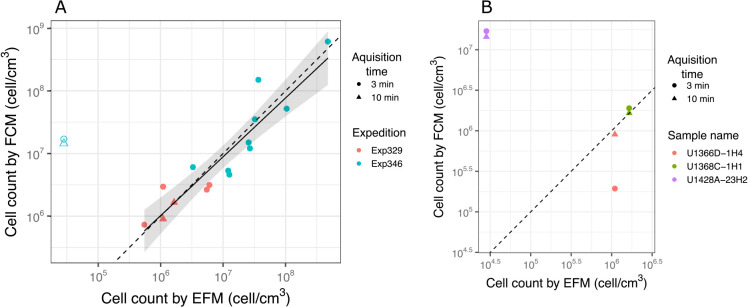
(A) Comparison of microbial cell counts by EFM and FCM. (B) Comparison of FCM acquisition times and staining methods. The black dashed line shows a 1:1 line for the counts by EFM and FCM. The black solid line shows a regression line: log cell count (FCM)=0.94 log cell count (EFM)+0.4, R^2^=0.86, *P*<0.00001. The shaded area indicates the 95% confidence interval. The open symbol in (A) indicates a sample with an EFM count <10^5^ cells cm^–3^, which was not included in the regression model.

We applied our validated in-liquid staining procedure to subseafloor sediments in which cell abundance ranged between 10^4^ and 10^8^ cells cm^–3^. To stably obtain FCM cytograms with distinguishable cells and non-cell particles, we stained cells in sediment suspensions with a ^v^SYBR/^v^Sed ratio of 10. FCM cell counts were consistent with manual and image-based microscopic counts from a range of 10^6^ to 10^8^ cells cm^–3^ (*R^2^*=0.86, *P*<0.00001, *n*=15, Fig. 3A), and the regression slope between FCM and EFM was close to the theoretical slope of 1:1 (slope=0.94), which is consistent with previous findings obtained using the on-membrane staining procedure (Morono *et al.*, 2013).

However, the abundance of cells measured by the FCM method sometimes deviated from the EFM count when we analyzed samples at approximately 10^6^ cells cm^–3^, with an inadequate number of cells being detected by FCM (<11‍ ‍cells, Table S2). A change in the acquisition time in FCM from 3 to 10‍ ‍min increased the number of cells, thereby providing more accurate results (Fig. 3B). However, abundant non-cell particles often hinder FCM measurements of samples with lower cell abundance by acquiring non-targeted events, as discussed later (*i.e.*, other than cells). Even though multiple acquisitions are possible per sample, measurements of cell abundance as low as 10^6^ may be a reasonable range in the total analysis time. In samples with lower cell abundance, cell separation (Morono *et al.*, 2013) will be necessary to reduce the relative abundance of non-cell particles over cells.

We detected unknown green signals during FCM analyses. The FCM counts of samples with EFM cell abundance of less than 10^5^ cells cm^–3^ were markedly higher, corresponding to 10^7^ cells cm^–3^ (Fig. 3A). EFM observations revealed green particles that were similar in size to microbial cells, but with a different color and shape (Fig. S2). Although they were visually distinguishable by EFM and the region of the signal slightly shifted from the cellular region, they did not fall into distinct regions in the FCM cytogram. To prevent false-positive increases in cell counts, anomalously high cell detection in one of the series of samples needs to be carefully examined for this issue. When we analyzed the blank control, we detected a signal in the cellular region in each run (Table S2), which was attributed to SYBR-I dye precipitates. In this case, we observed irregularly shaped, green fluorescent particles (Fig. S3). However, these noise signals were only observed 5–7×10^3^ times per 1‍ ‍mL of the blank control and, thus, are not a significant concern in FCM counts. The unexpected precipitation of SYBR-I and its heavy interference with FCM cell counting was previously reported (Morono *et al.*, 2009; 2011a). Collectively, these examples demonstrate the potential pitfalls of FCM counting, which need to be considered in order to consistently produce reliable counts.

## Conclusion

We herein developed a rapid and straightforward method for staining microbial cells in subseafloor sediment for a high-throughput cell abundance analysis using FCM. Although it has been challenging to achieve stable staining quality, we identified the SYBR-I dye and sediment ratio as a critical factor for method standardization. We also described a simple approach to confirm successful sample staining based on the color of the supernatants of stained suspensions. By applying a stable staining procedure, we obtained a distinguishable FCM cytogram between cells and non-cell particles, which allowed us to obtain reliable data without a labor-intensive process for subseafloor sediment samples (>10^6^ cells cm^–3^). However, in samples with <10^5^ cells cm^–3^, it is recommended to pre-concentrate microbial cells using a cell separation method because procedural noise interferes with the precise analysis of cell abundance.

## Citation

Mori, F., Nishimura, T., Wakamatsu, T., Terada, T., and Morono, Y. (2021) Simple In-liquid Staining of Microbial Cells for Flow Cytometry Quantification of the Microbial Population in Marine Subseafloor Sediments. *Microbes Environ ***36**: ME21031.

https://doi.org/10.1264/jsme2.ME21031

## Supplementary Material

Supplementary Material
