# Supplementary material for: Simple In-liquid Staining of Microbial Cells for Flow Cytometry Quantification of the Microbial Population in Marine Subseafloor Sediments
Source: Microbes Environ. 2021 Aug 24;36(3):ME21031. doi: 10.1264/jsme2.ME21031 (PMC8446754; doi:10.1264/jsme2.ME21031)
Supplement: Supplementary file 1 — Supplementary Material [file 36_21031_s1.pdf]

Supplementary materials for

Simple In-liquid Staining of Microbial Cells for Flow Cytometry Quantification of the Microbial Population in Marine Subseafloor Sediments

Fumiaki Mori [1], Tomoya Nishimura[1], Taisuke Wakamatsu[2], Takeshi Terada [3], and Yuki Morono [1*]

[1] Geomicrobiology Group, Kochi Institute for Core Sample Research, Japan Agency for Earth-Marine Science and Technology (JAMSTEC), Monobe B200, Nankoku, Kochi 783–8502, Japan

[2]Agricultural Science, Graduate School of Integrated Arts and Sciences, Kochi University, Monobe B200, Nankoku, Kochi 783–8502, Japan

[3] Marine Works Japan Ltd., Oppama-higashi 3–54–1, Yokosuka 237–0063, Japan

*** Corresponding Author.** Email: morono@jamstec.go.jp; Tel +81–88–878–2273

Table S1: Results of FCM analysis for in-liquid staining with various dye amount.

						All FCM event number					FCM event numbers when the beads count was adjusted to around 1500		
	FCM aquisition time	Sediment volume in the stained suspension (μL)	Amount of dye (μL)	Dye amount ratio (vSYBR/vSed)	Dye concentration at the staining process (×)	Detected total events in FCM	Counted beads in FCM	cells in FCM gate	FCM (cell/mL)		Detected total events in FCM	Counted beads in FCM	cells in FCM gate
Staining once with dye	3min	0.2	2	10	909	658445	1524	2884	503375328		647364	1494	2836
	3min	0.2	1	5	476	448139	1817	4048	592607595		531130	1497	2852
	3min	0.2	0.325	1.63	100	743556	2116	4059	510252363		530648	1506	2922
	3min	0.2	0.125	0.63	50	738532	2135	19824	2469875410		525540	1501	14150
	3min	0.2	0.02	0.1	10	534197	2086	83797	10685523490		386556	1495	61062
	3min	0.2	0.002	0.01	1	69011	2031	3029	396708026		52221	1497	2291
	3min	0.8	1	1.25	476	1500000	1045	9026	574381818		1500000	1045	9026
Re-staining with additional dye (1μL)	3min	0.2	0.325	1.63	100	890678	2046	3424	445153470		645383	1499	2491
	3min	0.2	0.125	0.63	50	925019	1980	3940	529313131		705571	1508	3018
	3min	0.2	0.02	0.1	10	855390	1997	3714	494704056		648512	1500	2796
	3min	0.2	0.002	0.01	1	848740	1596	3936	656000000		809118	1508	3757

Table S2: Results of FCM analysis for sediment samples with a variety of cell concentration

Exp	sample name	cells in FCM gate	Counted beads in FCM	Detected total events in FCM	FCM aquirsition time setting	Actual FCM aquirsition time (s)	FCM (cell/mL)	EFM (cell/mL)	EFM method
Exp329	U1366D-1H1	9	763	1500000	3min	76	3137615	6031115	Auto count using metamorph
Exp329	U1366D-1H4	1	1373	1359837	3min	193	193736	1096566	Auto count using metamorph
Exp329	U1366D-1H4	8	2358	1500000	10min	414	902460	1096566	Auto count using metamorph
Exp329	U1368C-1H1	11	1540	57358	3min	193	1900000	1644850	Auto count using metamorph
Exp329	U1368C-1H1	32	5146	149634	10min	613	1654100	1644850	Auto count using metamorph
Exp329	U1368C-2H2	6	2188	15421	3min	193	729433	548283	Auto count using metamorph
Exp329	U1371E-1H1	12	1079	1500000	3min	107	2958295	1096566	Auto count using metamorph
Exp329	U1371E-2H2	11	1105	1500000	3min	95	2647964	5482832	Auto count using metamorph
Exp346	U1428A-1H1	2714	1177	565635	3min	193	613359388	474613883	Auto count using metamorph
Exp346	U1428A-2H1	701	1242	1187268	3min	193	150133655	36734975	Auto count using metamorph
Exp346	U1428A-3H1	269	1378	776167	3min	193	51925980	104173810	Auto count using metamorph
Exp346	U1428A-4H2	181	1374	1130528	3min	193	35040757	32348709	Auto count using metamorph
Exp346	U1428A-5H1	77	1365	739788	3min	193	15005128	25609043	Auto count using metamorph
Exp346	U1428A-7H1	26	1300	1004406	3min	193	5320000	12062231	Auto count using metamorph
Exp346	U1428A-9H1	57	1254	951021	3min	193	12090909	26865877	Auto count using metamorph
Exp346	U1428A-12H1	22	1270	1458186	3min	193	4607874	12610514	Auto count using metamorph
Exp346	U1428A-15H1	32	1405	1500000	3min	160	6058363	3289699	Auto count using metamorph
Exp346	U1428A-23H2	103	1614	838878	3min	193	16975217	28314	Eye observation using EFM
Exp346	U1428A-23H2	220	4057	1500000	10min	546	14424452	28314	Eye observation using EFM
	blank (water)	7	2397	2588	3min		7000		
	blank (water)	5	1370	1540	3min		5000		

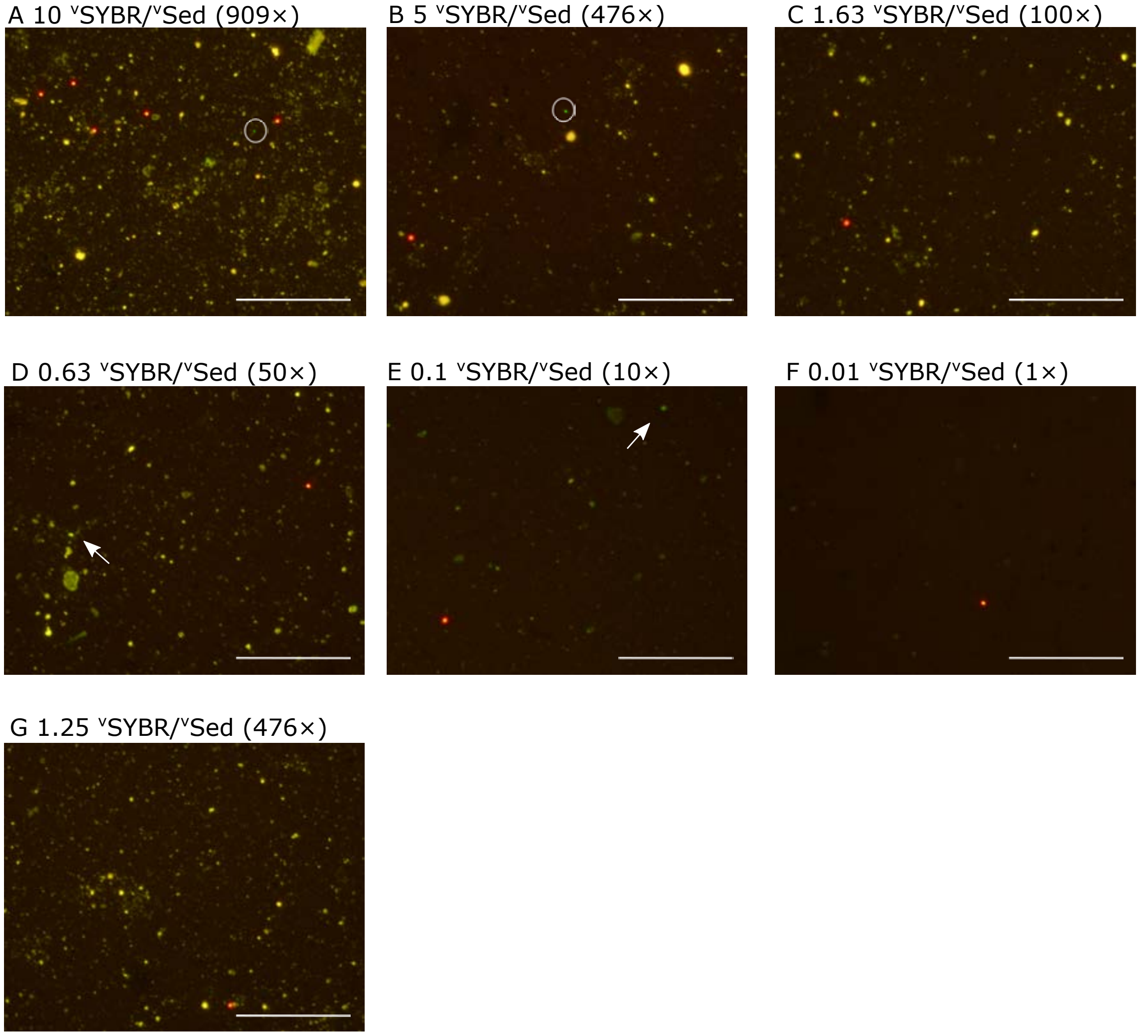

Fig. S1 Microscopic observation of the sediment used for the FCM analysis. Each sediment sample (U1428A-1H1) stained with the various amounts of SYBR-I [A: 10 ^{v}SYBR/^{v}Sed (909×), B: 5 ^{v}SYBR/^{v}Sed (476×), C: 1.63 ^{v}SYBR/^{v}Sed (100×), D: 0.63 ^{v}SYBR/^{v}Sed (50×), E: 0.1 ^{v}SYBR/^{v}Sed (10×), F: 0.01 ^{v}SYBR/^{v}Sed (1×), G: 1.25 ^{v}SYBR/^{v}Sed (476×)]. The same volume of sediment samples (0.2 µL of sediment in 20 µL of sedimentary suspension) were stained with various amount of SYBR-I, but four-time sediment volume (0.8 µL of sediment in 20 µL of sedimentary suspension) was stained for (G). The circle indicates stained cells with green fluorescence. The white arrow indicates greener non-cell particles. The red particles are beads. White bar: 50 µm.

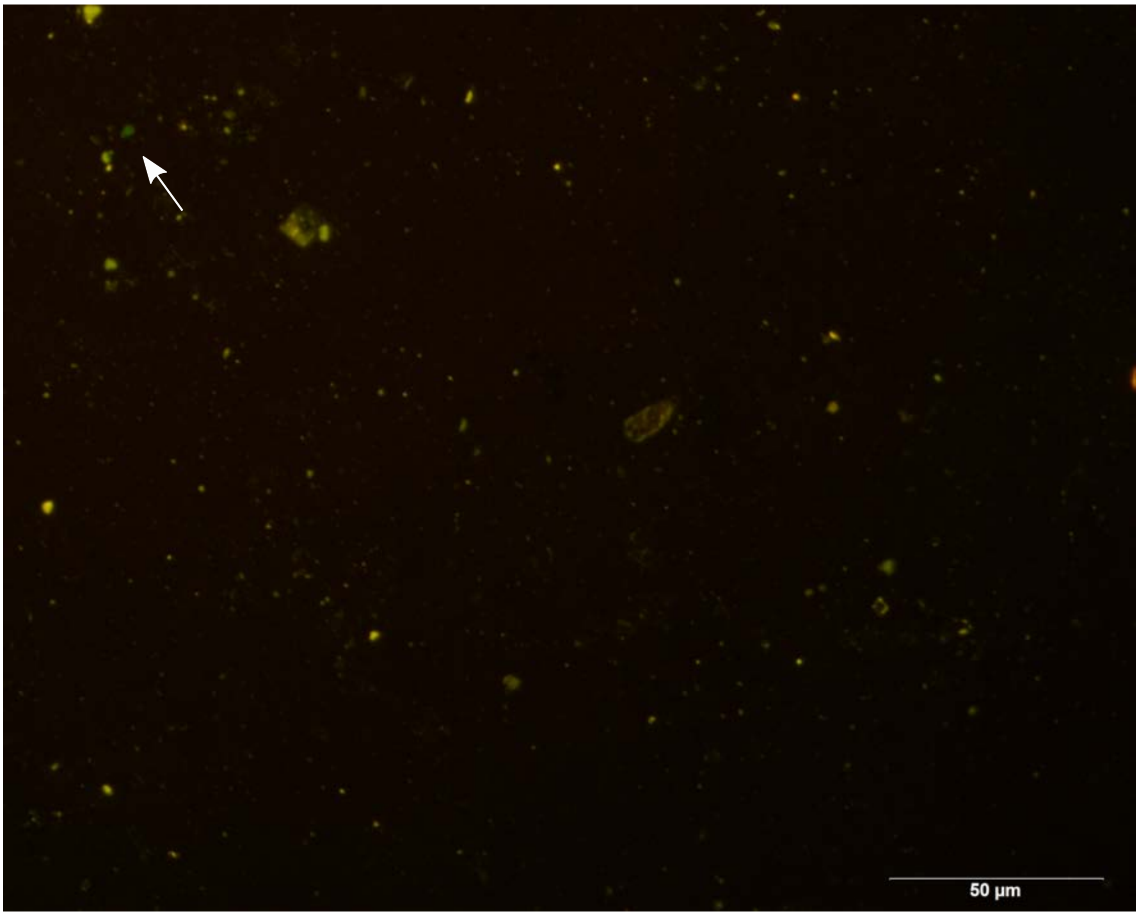

Fig. S2 Microscopic observation of the sediment sample (U1428-23H2) used for the FCM analysis. The sample was stained using in-liquid staining method (10 ^{v}SYBR/^{v}Sed). The white arrow indicates greener non-cell particles. Bar: 50 μm.

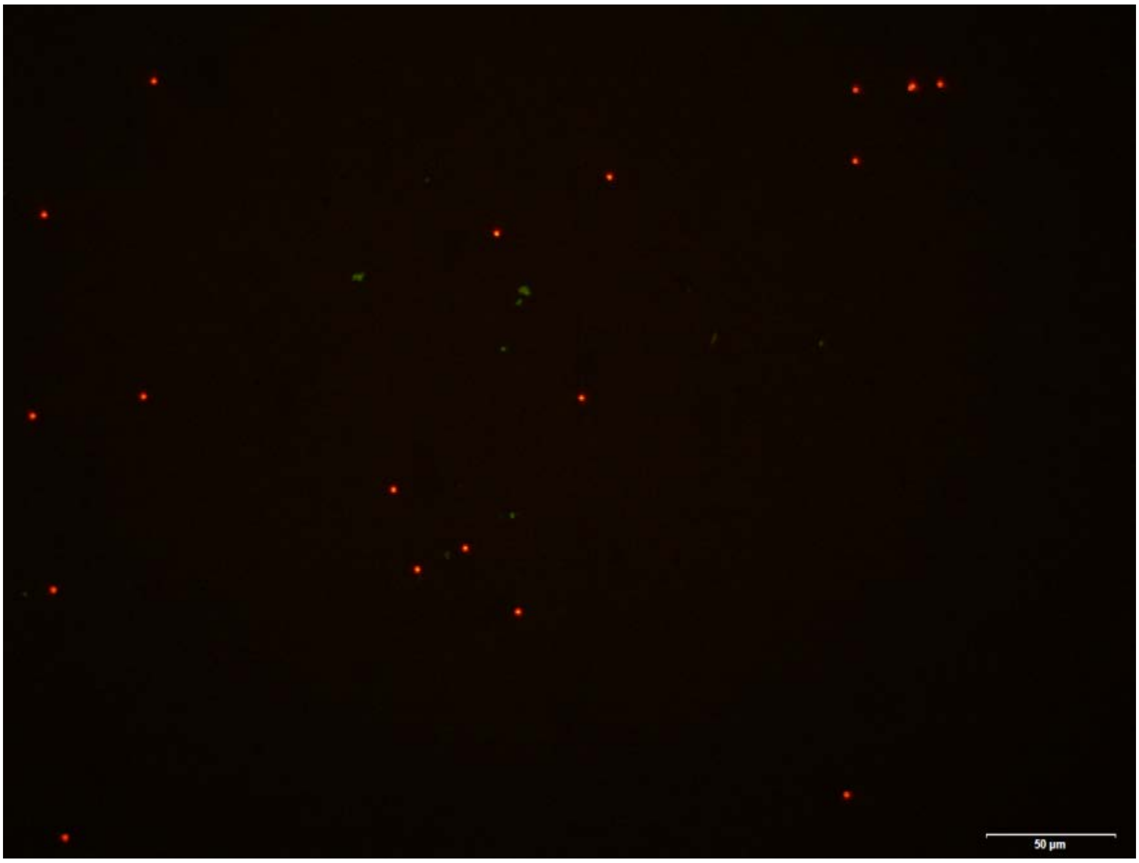

Fig. S3 Microscopic observation of the blank (filter sterilized water) used for the FCM analysis. The blank was stained with the SYBR-I (909×). The red particles are beads. All greener particles are non-cell. White bar: 50 µm.
